# Child maltreatment prevention: a content analysis of European national policies

**DOI:** 10.1093/eurpub/cky176

**Published:** 2018-09-03

**Authors:** Maria Ramiro-Gonzalez, Darja Dobermann, Dmytro Metilka, Emogene Aldridge, Yongjie Yon, Dinesh Sethi

**Affiliations:** Division of Noncommunicable Diseases and Promoting Health through the Life-Course, WHO Regional Office for Europe, Copenhagen, Denmark

## Abstract

**Background:**

Child maltreatment is a major public health concern, which worsens inequalities and perpetuates social injustice through its far—reaching impacts on the health and development of children affected. The aim of this article was to provide a content analysis of the national policies presently used to address child maltreatment and provide an overview of prevention practices being employed in countries of the World Health Organization (WHO) European Region. This analysis will aid in identifying, which policy areas still require further work to prevent child maltreatment.

**Methods:**

Three search methods were employed to identify national policies on child maltreatment. A framework based on WHO guidelines for the development of policies was used to conduct a policy analysis of the identified national policies.

**Results:**

Two hundred and seventy-eight national policies were identified; of these, 68 met the inclusion criteria for further analysis representing 75% of the WHO Europe Region Member States. Whereas the majority of policies fulfilled most of the WHO criteria for effective policy-making, only 34% had a budget and 6% had quantified objectives. Plans to implement proven child maltreatment prevention interventions were high, with the exception of some countries where the health sector is in the lead.

**Conclusions:**

The key policy areas requiring improvement were quantifiable objectives and allocated defined budgets. Hospital-based and home-based child maltreatment interventions were also not widely planned for implementation. Encouraging progress is being made on national policy development to prevent child maltreatment. There are as of yet, several key areas, which warrant increased attention in future policy-making.

## Introduction

Child maltreatment, defined as ‘all forms of physical and/or emotional or sexual abuse, deprivation and neglect of children or commercial or other exploitation resulting in harm to the child’s health, survival, development or dignity’, is responsible for at least 850 premature deaths a year in children under 15 in the European Region.^1^ It is likely that in reality, these numbers are even higher as many child deaths are not investigated and cases of maltreatment often go unrecognized.^2^ It is also estimated that for every child death, there are between 150 and 2400 cases of significant physical abuse.^3^ Survey data from the European Region indicate that approximately 23% of children experience physical abuse, 29% experience emotional abuse, and just over 13% of girls and almost 6% of boys experience sexual abuse.^4^

This and other types of early childhood adversity have been demonstrated to having lasting consequences throughout the life course. There is evidence of an increase in later risk-taking behaviour, such as smoking, alcohol misuse, drug-use and high-risk sexual behaviour, and an increase in the prevalence of disorders such as diabetes, obesity and depression.^5–7^ These behaviours and disorders are causally linked to major public health problems such as cardiovascular disease, liver cancer, autoimmune disease, sexually transmitted infections, interpersonal violence and suicide.^5^^,^^8^^,^^9^ Next to direct health outcomes child maltreatment is also associated with non-health outcomes such as decreased educational achievement, lost wages, perpetration of future violence or criminal activity and reduced quality of life.^5^^,^^10–12^

In addition to the direct negative consequences to the child, the cost of child welfare, child and adult health care, criminal justice costs and productivity loss as a consequence of child maltreatment place a preventable strain on social- and public health-systems. For example, in Germany and Italy the economic costs due to child maltreatment are estimated to be between 11 and almost 30 billion Euros annually.^2^^,^^13^

It is evident that child maltreatment has significant individual, societal and economic repercussions. Action to prevent child maltreatment is the best evidenced approach to reducing these repercussions from all angles^2^^,^^6^^,^^14–16^ and has led to the adoption of the World Health Organization (WHO) European Region, with a set of priorities and ‘Investing in children: the European child maltreatment prevention action plan 2015–2020’.^17^ This policy is framed within Health 2020 and its emphasis on equity, the life-course and public health approach.^18^ The prevention of child maltreatment features prominently in the Sustainable Development Goals (SDGs) with four targets (Targets: 5.2, 5.3, 16.1 and 16.2), which address ending violence against children and several targets (within Goals 1, 3, 4, 5, 10, 11 and 16), which address risk factors.^19^

With this amplified focus on child maltreatment countries are increasingly developing new or amending existing national policies targeted at child maltreatment. Policies have the potential to bring together multiple sectors to achieve set objectives, but only if they are visionary and well-developed.^20^ Policy development is also a significant step in the translation of research into practice and ultimately legislation.^21^ In line with the goals of these health agendas and the shifting policy landscape, this article aims to provide a content analysis of European national policies on child maltreatment, with an additional focus on prevention interventions, to identify any areas for improvement, while policies continue to be developed. This content analysis is done in the context of the WHO European Region and utilizes a framework based on the WHO guidance for identifying successful policy development.^21^

## Methods

For this article the ‘policy’ definition used in the WHO guide was adopted: ‘a document that sets out the main principles and defines goals, objectives, prioritized actions and coordination mechanisms’ and ‘provides the basis for action to be taken jointly by the government and its non-governmental partners’.^21^ Additionally, the terms ‘action plan’ or ‘strategy’ or ‘programme’ can be used to designate a policy document.^22^^,^^23^ As such national documents titled with any of these alternative terms were included if they met the above policy definition.

### Collation of documents

Three approaches were used to collect national policies on child maltreatment

1. Responses to the 2014 Global Status Report on Violence Prevention questionnaire from the National Data Coordinators from each European Region Member State, regarding the existence of national policies on child maltreatment, were reviewed.^24^
2. Internet-based search of the websites of European ministries of health, justice, education, youth, social security, social affairs, human rights and equality to identify policies.
3. A ‘Google’ search in English (to identify any policy documents, which may have been missed), using the following key words: child, children, maltreatment, abuse, national, action plan, strategy, program, protection, prevention and Europe.

All available versions of documents were collected (*n* = 278) for the period January 2000 to December 2016 and filtered to select out national policies directly related to child maltreatment (*n* = 226). Documents obtained, which did not meet the criteria of national policy such as: national recommendations or guidelines, legislation and regulation, progress reports on national policies and general knowledge reports were excluded at this stage. ‘Google translate’ was used to analyse documents not written in English. If the search returned multiple versions of the same policy only the most recent versions of publications was utilized. The oldest policy was from 2000. All policies since 2000 were examined and only policies which also contained child maltreatment prevention practices were included as evidence has shown investment in prevention practices to be more effective than only addressing the consequences of child maltreatment.^4^^,^^25^^,^^26^

### Analysis of documents

A proforma was developed based on criteria that have been previously described for policy frameworks in WHO guidance by Schopper *et al.*^21^ and Gray *et al.*^23^ This framework, consisting of nine variables (table 1) adapted from and based on the WHO guide to analysing the comprehensiveness of national policies on child maltreatment was used initially. Such a framework has been previously successfully used to conduct a content analysis of European national policies addressing violence and injury prevention.^27^ The percentage of national policies, which met each of the criteria was calculated using eligible national policies as the denominator.

**Table 1:** Framework to conduct content analysis of policies and preventive interventions analysed in secondary policy analysis

Policy content analysis framework	
Variables	Descriptions
Quantified objectives	Reduction in cases of child maltreatment by a quantified amount over a defined time period
Time frame	Clear time frame defined for implementation of the policy
Target population	Clearly defined population groups targeted by the policy such as children and or families either in the general population or those at higher risk
Multi-sector involvement	Participation of different stakeholders from different sectors such as health, welfare, justice, education and non-governmental organizations in formation and implementation of the policy
Planned interventions	Preventive interventions to be implemented to address specified objectives
Lead agency	Specified public administrative body, which is responsible for the development, coordination of implementation and policy outcome evaluation
Budget	A budget to finance policy development and implementation is mentioned or implied within the document
Monitoring and evaluation	Mechanism in place or in development for monitoring policy implementation process and evaluating its effectiveness in the target population in achieving specified objectives
Government minister/ ministry approval	Formal approval by government, or government minister/ministry for policy development
**Preventive interventions analysed within national policies**	
Interventions	Descriptions
School-based violence prevention	Education programmes, based within schools, teaching children to recognize harmful situations and distinguish between appropriate and inappropriate forms of touch and include multicomponent preschool violence prevention programmes and sexual abuse training programmes such as Kidpower, Stay Safe
Public awareness	Programmes to disseminate messages on child maltreatment among the general population using channels such as: television, radio, social media and other internet platforms raising awareness of the issue, changing social norms regarding the acceptance of abusive behaviour, gender equality and encouraging reporting of maltreatment
Hospital-based programmes	Programmes in which health-care professionals educate new parents in the dangers of shaking their child and providing alternative strategies for dealing with persistent crying such as the Period of Purple Crying and the Shaken Baby Prevention Project
Home-visiting programmes	Programmes which provide intensive, in-home early years support for parents whose children are at risk of poor outcomes such as the Nurse Family Partnership, Early Head-start and Step towards Effective Enjoyable Parenting
Parenting programmes	Programmes to strengthen the relationship between parents and children and improve parents’ skills, knowledge and confidence to support child development and behaviour management such as Triple P (Positive Parenting Program), Parents Anonymous, Incredible Years, Adults and Children Together Against Violence and Parenting for Lifelong Health
Capacity development	Programmes designed to increase the skills and confidence of health-care staff and other professionals for identifying and preventing child maltreatment such as Safe Environment for Every Kid
Community interventions	Programmes to enhance community capacity to prevent child maltreatment by expanding resources and promoting a culture of collective responsibility for positive child development and include support and mutual aid groups for parents, early child care services, care of vulnerable children and improving residential care services
Legal action	Specific laws for prohibiting child maltreatment and reducing its risk factors, along with clear courses of action (e.g. fines, penalties, sentences) to be taken when laws are violated and include those against corporal punishment in all settings, intimate partner violence, sexual abuse and exploitation and labour exploitation

Subsequently, analysis was done to identify the implementation rate of interventions to prevent child maltreatment within the WHO European Region Member States. A proforma was used to examine eight specific interventions (table 1). These had been previously established as cornerstone interventions for successfully preventing child maltreatment in comprehensive reviews.^4^^,^^16^^,^^25^^,^^28^^,^^29^ This analysis was done by country rather than by policy. The interventions in this context encompassed all three levels of prevention, universal, targeted and indicated.^26^ Data was obtained and checked by two reviewers (M.R.G. and E.A.); the first collated the publications and extracted key items for analysis and the second cross-checked these for accuracy and completeness. Any disagreements were resolved by consulting a third reviewer (D.S.). Data entered into spreadsheets were cross-checked against the national policies and analysed using the Excel Analysis Toolpak.

## Results

**Figure 1:**
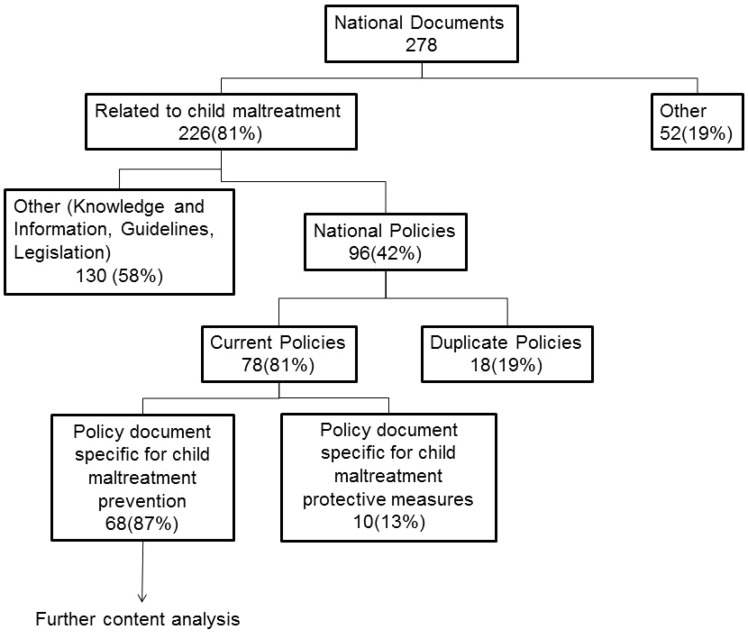
Flow-chart identifying the collected documents on national policies about child maltreatment (as of December 2016)

Figure 1 shows a summary of all the national documents collected until December 2016. A total of 278 documents were obtained, of which 226 pertained to child maltreatment and 52 did not. Ninety-six out of the total of 278 documents were identified as national policies, 78 of which were current policies, and 18 of which were duplicate policies. Sixty-eight out of the 78 documents were identified as focused on prevention, representing 40 countries of the 53 in the WHO European Region. Out of these 40 countries for which child maltreatment policies were identified 23 (58%) were European Union (EU) countries, and 17 (42%) were non-EU countries that belonged to the WHO European region.

### Content analysis of 68 European policies on child maltreatment

**Figure 2:**
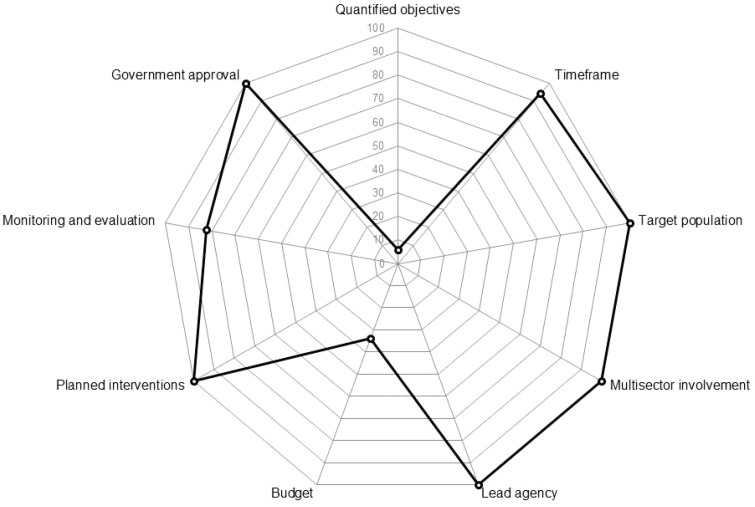
Percentage of national policies, which met criteria of the policy framework (table 1)

Figure 2 shows the results of the general content analysis of the 68 European policies using the variables described in the policy analysis framework (table 1).

Multi-sectoral collaboration, government minister/ministry approval and a lead agency were mentioned in all of the policies. Further all national policies also stated definitive planned interventions to achieve their objectives. However, while all of the national policies had general objectives for child maltreatment prevention, only 6% of the policies had quantified objectives with targets to be achieved over a specific time frame. For example, Finland set the goal to reduce the number of children experiencing corporal punishment by 50%.^30^ Despite the overall lack of quantified objectives, the majority of the policies (82%) demonstrated clear intent to monitor and evaluate the implementation and effectiveness of policies and interventions. Only a minority of policies (34%) indicated a budget for the policy implementation and evaluation; 66% of policies did not specify financial support.

Ninety-four percent of the national policies specified a time frame within which interventions should be implemented and/or objectives achieved. Time frames for implementation ranged from 1 year (Bosnia and Herzegovina, ‘Action plan for child protection and prevention of violence against children through information-communications technologies in Bosnia and Herzegovina 2014–2015’) to 13 years (the United Kingdom, ‘The Children’s Plan: Building brighter futures’), with the median time frame being 4 years. Further, all of the national policies that were analysed had a defined target population, ‘children’. Within this, almost 80% of the policies were targeted specifically at children and families considered to be at risk of maltreatment, while 91% of the policies also included the wider public (e.g. people working with children, communities, NGOs etc.) as their target population.

### Analysis of interventions in 40 WHO European Region Member State countries

**Figure 3:**
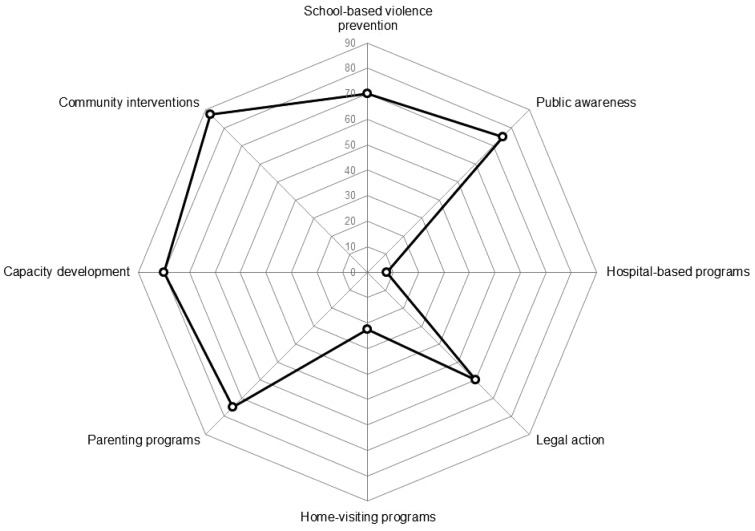
Implementation rate of interventions to prevent child maltreatment (table 1) in 40 WHO European Region Member State countries

Figure 3 shows the implementation percentages of the core child maltreatment prevention interventions (table 1) within the 40 WHO European Region Member State countries identified by the 68 national policy documents on child maltreatment.

The majority of interventions were found in over 70% of the countries. Thirty of the 40 countries had parenting programmes and public awareness efforts within their policy. While 28 had school-based violence prevention programmes in place. Thirty-two of the 40 had some form of capacity building training in place for individuals working with children, and 35 of 40 also had community interventions to help build collective responsibility for child welfare.

The two least implemented interventions were hospital-based programmes and home-visiting programmes with only 3 and 9 out of 40 countries having them in place, respectively. A distant third lowest to these two interventions is legal action with only around half of countries (24 out of 40) including legal action within their policies.

## Discussion

This is the first content analysis of child maltreatment prevention policies in the WHO European Region. Sixty-eight policies from 40 countries were identified and utilized in the analysis. This quantity of recent policy documents is an encouraging step towards tackling the wider ramifications of child maltreatment. Further, unlike in other health policy analysis papers^27^ where patterns of policy distribution were identifiable (e.g. few policies from the Eastern part of the region), further analysis found no discernible patterns based on income, EU/non-EU or geographic location. This suggests that most countries across the region are aware of the importance of policies to prevent child maltreatment and more focus can be shifted to the content and comprehensiveness of policies to increase their effectiveness. Greater advocacy and policy development is needed in those countries where policies were not identified.

The lack of clearly defined budgets and quantified objectives were the biggest hurdles identified in the national policies analysed, present in only 34% and 6% of national policies, respectively. Ambiguous or non-existent budgets within a national policy suggest that no specific funding has been made available for actions within the policy. Without access to adequate resources interventions and strategies will struggle to be implemented increasing the risk that the policy will fail. A lack of quantified objectives also carries a risk. Previous guidelines suggest that having quantified objectives or targets contributes to the coherence of the policy and makes it easier to evaluate its effectiveness, making it more likely to succeed.^21^ Additionally, the majority of the national policies (82%) indicated intent to monitor and evaluate the implementation and effectiveness of the policy. The lack of measurable objectives or targets will present a significant hurdle to these countries in measuring, in concrete terms, the effect or implementation level of their policy. A lack of measurable outcomes also means there is a less clear way for a country to clearly demonstrate that it has made improvements; quantifiable objectives provide a platform for accountability as emphasized in Investing in children: the European child maltreatment prevention action plan 2015–2020 and the SDGs.^17^^,^^19^ Many countries lack reliable surveillance systems for child maltreatment, and there may therefore be a reluctance to incorporate objective measures in such policies.^4^^,^^31^ The importance of effective surveillance has been emphasized as key to monitoring violence prevention interventions and policies to ensure the fulfilment of the United Nations Convention on the Rights of the Child.^4^^,^^25^^,^^32^

The further analysis of the specific interventions mentioned in the national policies revealed home-visiting programmes and hospital-based programmes to be the most neglected interventions, with less than 23% and 8% of the countries implementing them, respectively. There was no identifiable relationship between the countries that chose to employee these interventions and those who choose not to. These finds are congruent with global findings with only 23% of countries having large scale home-visiting programmes and a further 35% having limited programmes.^24^ It is noteworthy that the two programmes, which are least implemented are also the programmes, which traditionally fall under the remit of the health sector; which has to date not necessarily considered prevention actions to be a part of their remit.^21^^,^^27^ In fact of the 68 policies analyzed only 11 (16%) included ministries of health as lead agencies on policies. This finding highlights the continued necessity of involving the health sector in prevention activities, at the policy and programmatic level. It is not to say that the health sector must take the lead on these activities as they are one of many bodies with a vested interest in preventing child maltreatment but they must be engaged in the activities. Another intervention that needed improvement is legal action, which is critical to enforcing comprehensive laws to protect children and modifying community and parenting behaviour such as violent discipline.^28^^,^^29^^,^^32^

The data to support the implementation of hospital-based training of parents to prevent abusive head trauma clearly shows significant reductions in abusive head injuries in infants and young children from shaking.^33^ Such training may also be applied in community settings. Research on home-visiting programmes on the other hand has produced mixed results, with few trials showing them to directly significantly reduce or prevent child maltreatment.^34^^,^^35^ However, while home-visit programmes may not show evidence of directly reducing child maltreatment the research strongly supports that they improve parental skill and knowledge, support positive cognitive and social development of children, and strengthen the link between parents and other social or health care services; thereby reducing the risk factors for child maltreatment.^16^^,^^36^

While both programmes are initially resource intensive cost-benefit analyses show the benefits of the programmes outweigh the costs of implementation.^4^ For example, a cost-benefit analysis of a meta-analysis of home-visiting programmes in the US found that, on average, for every 1 USD spent there was a saving of 2.24 USD over time. Additionally, the higher the risk group targeted by the programme the higher the savings tended to be, reaching as high as 5.70 USD for the highest risk groups, defined in the study as low socio-economic, single parents.^37^

Another common area of failure in implementation of interventions is the lack of integration of the programme with existing health and social care services. In the case of home visitors, they are meant to serve as the bridge between families and other professional services and community resources, using a multi-sectoral approach to achieve the best outcome.^35^ Categorical, ‘siloed’ approaches which are competitive, with each entity trying to preserve what is ‘theirs’, rather than collaborative approaches doom interventions to fail.^38^ Effective intervention programmes for child maltreatment must be based on close interactions between all national institutions, such as Health, Welfare, Education and Law, as well as any public programmes and systems already in place, who can play pivotal roles within communities raising awareness.^2^ Forming multi-sectoral partnerships and cooperating within them is well-evidenced as vital to attaining successful outcomes; it is necessary to act simultaneously across several different levels to prevent violence.^26^ Health 2020, the SDGs, and the INSPIRE package all reiterate the necessity of a collaborative approach to getting public health priorities into high-level polities and having the greatest impact.^18^^,^^19^^,^^28^

The findings of this article must be considered in light of several limitations. The inability to search for or analyse non-English policies inherently means some policies will have been excluded limiting the generalizability of the findings. Google translate was used to mitigate the impact of this. Due in part to this and other potential search limitations it is possible the data is under-representative of the WHO European Region, though the policies did cover 75% of the WHO Europe Region Member States. Lastly, it is acknowledged that several model frameworks for content analysis exist^39^^,^^40^ thus the choice to use the framework developed by Schopper *et al.*^21^ may be a limitation. However, this framework was chosen as it has been specifically developed for use with policy development and combines aspects of other frameworks resulting in a more comprehensive content analysis.^27^ Similarly, the analysis of prevention interventions was limited to their identification in national policy, without consideration of breadth and intensity of implementation, nor fidelity to programme design.^29^ It is also possible that there may be preventive interventions that are being implemented in countries but which are not mentioned in existing national policies. To accommodate for this all policies between were 2000 and 2016 were examined. However, the remit of this article is a content analysis of national policies and future research could also include triangulation with other sources.^24^

## Conclusion

To conclude, this article has shown that progress is being made in the region in developing national policies on child maltreatment. The majority of the policies from the 40 WHO Europe Region Member States analysed fulfilled most criteria set forth by the policy framework. The framework, which was developed for this study will hopefully serve to facilitate further research in the future. This analysis had identified that most national policies are still lacking quantified objectives and clearly allocated budgets. Incorporating these two elements should be a priority for moving forward. Additionally, the increased implementation of evidence-informed interventions through inclusion in national policy is vital to reducing child maltreatment across the region. It is hoped that the short comings presented in this article will help countries to develop and implement more comprehensive national policies in child maltreatment prevention.

## Funding

M.R.G., a Public Health Specialist in the Admission Department of Gregorio Marañón General University Hospital, was able to undertake her internship at WHO to work on this article thanks to her Public Health Medical Residency. D.D. was able to undertake her internship at WHO to work on this article thanks to her Doctoral Training Partnership funded by the Biotechnology and Biological Sciences Research Council. D.M. was able to undertake his internship at WHO to work on this article thanks to his Phoenix Erasmus Mundus Joint Doctorate Programme on Dynamics of Health and Welfare funded by the European Commission.

## Disclaimer

The named authors alone are responsible for the views expressed in this publication and they do not necessarily represent the views, decisions or policies of the World Health Organization.

The mention of specific companies or of certain manufacturers products does not imply that they are endorsed or recommended by the World Health Organization in preference to others of a similar nature that are not mentioned. Errors and omissions excepted, the names of proprietary products are distinguished by initial capital letters.

*Conflicts of interest*: None declared.

**Key points**

- Forty of the 53 WHO Europe Region Member States were found to have policy documents on child maltreatment prevention.
- The majority of policies lack quantified objectives and clearly defined or allotted budgets.
- The majority of child maltreatment interventions are being implemented, however, interventions traditionally falling under the remit of the health sector, such as hospital-based training and home-visiting programmes, are still lacking in uptake.
- Future policies must strive to include quantified objectives and clear budgets along with increasing multi-sectoral integration of interventions with all child maltreatment stakeholders.
